# Interaction between *SIDT2* and *ABCA1* Variants with Nutrients on HDL-c Levels in Mexican Adults

**DOI:** 10.3390/nu15020370

**Published:** 2023-01-11

**Authors:** Guadalupe León-Reyes, Anna D. Argoty-Pantoja, Berenice Rivera-Paredez, Alberto Hidalgo-Bravo, Yvonne N. Flores, Jorge Salmerón, Rafael Velázquez-Cruz

**Affiliations:** 1 Genomics of Bone Metabolism Laboratory, National Institute of Genomic Medicine (INMEGEN), Mexico City 14610, Morelos, Mexico; 2 Research Center in Policies, Population and Health, School of Medicine, National Autonomous University of Mexico (UNAM), Mexico City 04510, Morelos, Mexico; 3 Department of Genetics, National Institute of Rehabilitation (INR), Mexico City 014389, Morelos, Mexico; 4 Epidemiological and Health Services Research Unit, Morelos, Mexican Institute of Social Security, Cuernavaca 62000, Morelos, Mexico; 5 Department of Health Policy and Management and Kaiser Permanente Center for Health Equity, Fielding School of Public Health, Los Angeles, University of California, Los Angeles, CA 90095, USA; 6 Cancer Prevention and Control Research Center, Fielding School of Public Health and Jonsson Comprehensive Cancer Center, University of California, Los Angeles, CA 90095, USA

**Keywords:** hypoalphalipoproteinemia, gene–gene interaction, gene–diet interaction, rs17120425, rs1784042, rs9282541, Mexican population

## Abstract

Previous studies have reported that the *SIDT2* and *ABCA1* genes are involved in lipid metabolism. We aimed to analyze the association—the gene x gene interaction between rs17120425 and rs1784042 on *SIDT2* and rs9282541 on *ABCA1* and their diet interaction on the HDL-c serum levels—in a cohort of 1982 Mexican adults from the Health Workers Cohort Study. Demographic and clinical data were collected through a structured questionnaire and standardized procedures. Genotyping was performed using a predesigned TaqMan assay. The associations and interactions of interest were estimated using linear and logistic regression. Carriers of the rs17120425-A and rs1784042-A alleles had slightly higher blood HDL-c levels compared to the non-carriers. In contrast, rs9282541-A was associated with low blood HDL-c levels (OR = 1.34, *p* = 0.013). The rs1784042 x rs9282541 interaction was associated with high blood HDL-c levels (*p* = 3.4 × 10^−4^). Premenopausal women who carried at least one rs17120425-A allele and consumed high dietary fat, protein, monounsaturated, or polyunsaturated fatty acids levels had higher HDL-c levels than the non-carriers. These results support the association between the genetic variants on *SIDT2* and *ABCA1* with HDL-c levels and suggest gene–gene and gene–diet interactions over HDL-c concentrations in Mexican adults. Our findings could be a platform for developing clinical and dietary strategies for improving the health of the Mexican population.

## 1. Introduction

Epidemiological studies have reported that low high-density lipoprotein cholesterol (HDL-c) levels are a principal risk factor for type 2 diabetes (T2D), obesity, stroke, metabolic syndrome (MetS), and cardiovascular disease (CVD) [1]. HDL-c is the primary vehicle for cholesterol transport from the peripheral cells to the liver for excretion and catabolism through reverse cholesterol transport (RCT). HDL-c exerts properties that confer protection to the cardiovascular system, including antioxidant enzymes, vasodilators, antithrombotic, immunomodulatory, anti-inflammatory, and anti-atherosclerotic effects [2]. The Mexican National Health and Nutrition Survey (ENSANUT, by its Spanish acronym) has consistently confirmed that the Mexican population has an alarmingly high prevalence of dyslipidemias [3]. Specifically, hypoalphalipoproteinemia is highly prevalent, affecting more than 50% of adults, with differences noted by sex and age groups [3,4,5]. HDL-c concentrations are strongly influenced by lifestyle habits, such as diet [6] and genetic factors [7]. Among the main causes are smoking, alcohol consumption, an unhealthy diet (intake of sugary drinks and high-carbohydrates and fat diet), sedentarism, drugs intake (e.g., steroids, statins, and fibrates) or other metabolic disorders (e.g., insulin resistance and liver disease) [6,7,8]. The impact of genetic factors has been evidenced through studies on twins, which have estimated that the heritability of low HDL-c levels ranges from 40% to 60% [7]. Therefore, numerous genome-wide association studies (GWAS) have identified more than 175 loci involved in lipoprotein metabolism [9]. However, most of these studies have been performed in the European populations [10,11], where just only a few investigations have explored these loci in the Mexican population. Lately, rs2000999 on the *HP* gene [12]; rs1784042, rs17120425 on *SIDT2* [13,14]; rs2975762 on *CAPN10* [15]; rs1800588 on *LIPC* [16]; rs224534 on *TRPV1* [17]; rs2278426 on *ANGPTL8* [18] and rs628031, rs594709 on the *SLC22A1* gene [19] have shown to be relevant in the HDL-c metabolism from Mexican populations.

The SIDT1 transmembrane family member 2 (*SIDT2*) gene, located on chromosome 11q23.3, which encodes to the SIDT2-lysosomal membrane protein [20], has been associated with lipid metabolism, mainly with triglycerides, HDL-c and the total cholesterol plasma levels in Asian populations [10,21,22]. Recently, we found that the allele-A of the intronic single nucleotide polymorphism (SNP) rs1784042 on *SIDT2* is associated with MetS through low HDL-c levels in the Mexican population [13]. We also reported that allele A of the missense variant rs17120425 (Val636Ile) on *SIDT2* increases the risk of T2D by reducing HDL-c levels [13], and these findings were confirmed by an independent analysis [14].

The ATP-binding cassette transporter A1 (*ABCA1*) gene, located on chromosome 9q31.1, promotes the efflux of free cholesterol to apoprotein A (apo A), which is critical for HDL-c biogenesis [23]. The missense polymorphism rs9282541 (alleles G/A) within *ABCA1* is relevant for the Mexican population because the rs9282541-A allele is almost exclusive to Native American people (~4%), while on other continents, it has not been found. The rs9282541-A allele has been associated with decreased HDL-c levels and cholesterol efflux in vitro (~27%), obesity, CVD, and T2D in the Mexican population [24,25,26,27]. Nevertheless, the pathophysiological mechanisms of these loci associated with the variations in HDL-c concentrations remain unknown. Recent studies have demonstrated that ethnic diversity may introduce differences in the frequency of risk alleles, leading to a potential predisposition to develop diseases or an altered response to treatment [28]. Additionally, it has been reported that dietary factors, such as protein, carbohydrate, and fat consumption, might modify the effect of several genetic variants associated with HDL-c metabolism, reporting significant gene–nutrient interactions [29,30]. For example, epigenetic studies have described that lipid- and carbohydrate-diet excess may elevate acetyl-CoA, which changes the chromatin structure and increases the acetylation and methylation DNA-sites for genes such as *APOE, IL6*, and *ABCA1*, which are correlated with CVD traits [31]. Additionally, hypercaloric diets increase DNA methylation in adipose tissue, especially in the promoter regions affecting the adipocyte differentiation, lipid metabolism, and adipose tissue expandability pathways [32].

Therefore, we might hypothesize that the interaction between the SNPs of the *SIDT2* and *ABCA1* genes could confer variation in the blood HDL-c levels in individuals carrying the lower-frequency alleles. Additionally, carriers of these variants—and those who consume diets rich in saturated fats and carbohydrates—could have decreased levels of HDL-c in plasma, unlike the individuals that consume hypocaloric diets. This knowledge will be helpful for identifying people at high risk of developing metabolic disorders or CVD. This study aimed to evaluate the potential effect of the interaction between the polymorphisms rs1784042 and rs17120425 on *SIDT2* and rs9282541 on *ABCA1* with HDL-c serum levels, and to analyze whether nutrients could modulate the impact of these SNPs in a cohort of Mexican adults.

## 2. Materials and Methods

### 2.1. Study Population

This study was a cross-sectional study that examined data obtained from the participants of the Health Workers Cohort Study (HWCS). The HWCS is a Mexican mestizo population-based cohort study focusing on the influence of genes and environmental factors on chronic diseases. The cohort design has been previously described in detail [33]. Briefly, the HWCS participants are medical, academic, and administrative employees from the Mexican Social Security Institute (IMSS, by its Spanish acronym), located in Cuernavaca, Morelos. Data were collected in the second wave of the sample collection, from 2010 to 2012. From the 2001 individuals enrolled in this study, We excluded 19 participants with missing clinical data. The remaining 1982 participants were included in the analysis.

This research was performed following the Declaration of Helsinki. The study protocol and informed consent form were approved by the Research and Ethics Committee from the IMSS (No. 12CEI 09 006 14). Informed consent was obtained from all participants.

### 2.2. Dietary Assessment

Dietary data were collected using a 116-item semi-quantitative food frequency questionnaire (FFQ); its validity and reliability have been previously reported [34]. The reported frequency for each food item was converted to a daily intake. Food composition tables, compiled by the National Institute of Public Health, were used to determine the nutrient compositions of all foods [33].

### 2.3. Genomic DNA Extraction and Genotyping

Genomic DNA was extracted from peripheral blood using a commercial isolation kit (QIAGEN systems Inc., Valencia, CA, United States of America) according to the manufacturer’s instructions. The variants rs1784042 (C_7500852_10) and rs17120425 (C_25922130_30) on *SIDT2* and rs9282541 (C_11720861_10) on *ABCA1* were genotyped using predesigned TaqMan SNP Genotyping assays (Applied Biosystems, Massachusetts, MA, USA) in a QuantStudio 7 Flex Real-Time PCR system (Applied Biosystems, Massachusetts, MA, USA). The automatic variant call was carried out with the SDS software, version 2.2.1.

### 2.4. HDL-c Levels

Blood samples were collected following a fasting period of at least 8 hours and then analyzed for HDL-c levels using an enzymatic colorimetric direct method (Synchron CX analyzer, Beckman Systems). According to the Adult Treatment Panel III (ATP-III) recommendations, low HDL-c levels were defined as HDL-c < 40 mg/dL in men or < 50 mg/dL in women [35].

### 2.5. Covariates

Demographic and lifestyle factors were evaluated using data obtained from the self-administered questionnaires. Leisure time of physical activity was assessed through a validated physical activity questionnaire that included 16 activity items, such as walking, running, and cycling. T2D was defined as having any of the following criteria: a medical history of T2D, currently taking T2D medication, and a plasma glucose level of >126 mg/dL after fasting for 12 h [36]. Hypertension was defined as systolic blood pressure of ≥140 mmHg or a diastolic blood pressure of ≥ 90 mmHg, based on the mean of two measurements obtained with a 5-min interval between them or if a hypertensive medication was reported. All participants were weighed using a digital scale (model BC-533; Tanita), and their heights were measured with a stadiometer (Seca). Body mass index (BMI) was calculated as weight [kg)/height (m)^2^], according to the World Health Organization criteria [36].

### 2.6. Statistical Power of the Study

The statistical power was estimated using the Quanto software (Department of Preventive Medicine, University of Southern California, Los Angeles, CA, USA) [37]. The dominant model of inheritance for allele frequencies ranges from 0.28–0.30 for rs1784042, 0.06–0.10 for rs17120425, and 0.07–0.10 for rs9282541 were used. Additionally, we considered the odds ratio (ORs) ranges between 0.75–0.77 for rs1784042, 0.48–0.52 for rs17120425 and 1.41–1.34 for rs9282541. These parameters were derived from this study, the 1000 genomes project database, and data reported in the literature. The disease prevalence was estimated to be 60% at a significance level of 0.05.

### 2.7. Statistical Analysis

We coded the genotypes of the three SNPs under the dominant model (0:GG ancestral genotype and 1:GA + AA genotype) due to the low prevalence of the risk allele. The statistical difference between the genotypes was analyzed by the Chi-squared test for the categorical variables and the Mann–Whitney *U* test for continuous variables. Hardy–Weinberg Equilibrium (HWE) was estimated using Pearson’s Chi-square test for the three SNPs.

The association between the three SNPs and HDL-c was determined under both the dominant and additive genetic models using a linear and logistic regression adjusted for age, sex, physical activity, smoking status, BMI, physical activity, lipid-lowering medications, smoking status, T2D, and hypertension to reduce the occurrence of spurious results. We performed a logarithmic transformation of the HDL-c levels for linear regression to bring their distribution closer to normal. As appropriate, the gene–gene and gene–diet interactions on HDL-c (continuous and categorical) were evaluated by adding a multiplicative interaction term to the linear regression or logistic model. A residual model that indirectly adjusts for total energy using a residual was used in the gene–diet interactions analysis. We performed sex–nutrient interactions on HDL-c and gene–diet interactions with nutrients (protein, carbohydrates, fiber, total fat, saturated fat, monounsaturated fatty acids (MUFAs), polyunsaturated fatty acids (PUFAs), and genetic variants (rs1784042, rs17120425, and rs9282541). We explored the interactions in the total population, stratified by sex and menopausal statuses. All statistical analyses were performed using the Stata software (version 14.0, StataCorp, College Station, TX, USA).

## 3. Results

### 3.1. Population Characteristics and Genotype Frequencies

This study included 1982 participants, where 70% were female, and of them, 44.6% were premenopausal women (n = 616). The median age of the participants was 52 years, and the prevalence of hypoalphalipoproteinemia was 60.2% (data not shown). The demographic and clinical characteristics showed that the median low-density lipoprotein cholesterol (LDL-c), HDL-c, and total cholesterol levels were higher in women than in men (*p* < 0.05). On the contrary, carbohydrates, proteins, total fat, MUFAs, and PUFAs dietary intake were higher in males (*p* < 0.05) than in females. Regarding genotypes, we observed similar proportions in men and women carriers of the GA + AA genotype in the three SNPs (Table 1).

When the demographics data were categorized by the genotype of each SNP, we observed that the carriers of the rs1784042-A and rs17120425-A alleles had higher HDL-c levels compared to the non-carriers (44.8 mg/dL vs. 43.1 mg/dL, *p* = 0.002, and 47 mg/dL vs. 43 mg/dL, *p* < 0.001, respectively). On the other hand, carriers of the rs9282541-A allele had lower HDL-c serum levels (41.4 mg/dL vs. 44.1 mg/dL, *p* < 0.001) (Table 2).

Additionally, we observed that among the HWCS participants, the minor allele frequency (MAF) of the rs1784042, rs17120425, and rs9282541 SNPs were 0.30, 0.10, and 0.10, respectively. They are similar to those reported in the 1000 Genomes Project database (http://1000genomes.org) for the population with Mexican Ancestry in Los Angeles, California (MXL). All SNPs were in HWE (*p* > 0.05) (data not shown). The sample size (n = 1982) was large enough and robustly powered to detect associations with similar ORs identified in previous GWAS and candidate-gene studies. The statistical power of this study was >90% for both the rs1784042 and rs17120425 variants and >70% for the rs9282541 variant.

### 3.2. Association Analyses between the rs1784042, rs17120425, and rs9282541 Polymorphisms with HDL-c Levels

The association analysis indicated that the rs1784042-A and rs17120425-A alleles were associated with high blood HDL-c levels (OR = 0.77, 95% CI: 0.66–0.90, *p* = 0.001 and OR = 0.52, 95% CI:0.42–0.66, *p* = 5.4 × 10^−8^, respectively). In contrast, the rs9282541-A allele was associated with low HDL-c under the dominant and additive models (OR = 1.41, 95% CI: 1.09–1.81, *p* = 0.008 and OR = 1.34, 95% CI: 1.06–1.69, *p* = 0.013, respectively) (Table 3). These associations were supported by the linear regression model (rs1784042 β = 0.03, 95%CI: 0.01, 0.04, *p* = 5 × 10^− 4^; rs17120425 β = 0.07, 95%CI: 0.04, 0.09, *p* = 2.5 × 10^−7^ and rs9282541 β = −0.05, CI: −0.07, −0.02, *p* = 1.34 × 10^−5^) (Table S1).

After stratifying by sex, we found a significant association between the three SNPs and HDL-c levels under the dominant and additive models (*p* < 0.05) in the female group. The rs1784042-A and rs9282541-A alleles did not reach a significant association (*p* > 0.05) among males, and the rs17120425-A allele showed a marginal association (*p* = 0.023) (Table 4). The results from the quantitative analysis stratified by sex remained in the same direction for females. The rs17120425-A and rs9282541-A alleles were borderline significant for the male group (Table S2).

### 3.3. Conditional Analysis

The conditional analysis showed that the rs1784042-A allele was associated with high blood levels of HDL-c in the presence of the rs9282541-A allele (β = 0.027, *p* = 0.011). Under a dominant inheritance model, the rs17120425-A allele remained associated with high blood levels of HDL-c when both the rs1784042-A (β = 0.072, *p* = 2.09 × 10^−6^) and rs9282541-A alleles (β = 0.076, *p* = 3.90 × 10^−8^) were present. Similarly, the rs9282541-A allele conserved the association for hypoalphalipoproteinemia in the presence of the rs1784042-A and rs17120425-A alleles (β = −0.064, *p* = 3.14 × 10^−6^ and β= −0.066, *p* = 1.11 × 10^−6^, respectively) (Table S3).

### 3.4. Gene–Gene Interaction with HDL-c Levels

**Figure 1:**
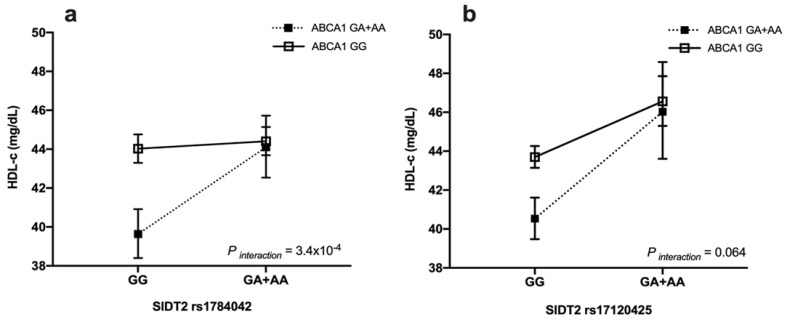
Interactions between polymorphisms of *SIDT2* and *ABCA1* on HDL-c levels in the total population. (**a**) *SIDT2*–rs1784042 x *ABCA1*–rs9282541 and (**b**) *SIDT2*–rs17120425 x *ABCA1*–rs9282541 interactions on HDL-c levels. HDL-c showed as mean, 95% confidence interval. Models adjusted for age (years), sex, body mass index (Kg/m^2^), physical activity (inactive/active), lipid-lowering medications (no, yes), smoking (no, current, past), diabetes (no, impaired glucose tolerance, yes), and arterial hypertension (SBP ≥ 140 mm/Hg or DBP ≥ 90 mm/Hg or antihypertensive drug).

We found that the carriers of at least one copy of the rs1784042-A and rs9282541-A alleles had high blood HDL-c levels compared to the non-carriers (OR = 0.40, 95%CI: 0.25–0.64, *p* = 1.4 × 10^−4^) (data not shown). Interestingly, the rs1784042 x rs9282541 interaction was statistically significant and showed an effect on the HDL-c levels under categorical (slope difference = 0.46, *p* _interaction_ = 0.003) (data not shown) and continuous (slope difference = 0.09, *p* _interaction_= 3.4 × 10^−4^) analysis (Figure 1a).

Additionally, the analysis showed that the carriers of at least one copy of the rs17120425-A and rs9282541-A alleles had high blood levels of HDL-c (OR = 0.28, 95% CI:0.16–0.49, *p* = 8.5 × 10^−6^), with respect to the non-carriers. However, the rs17120425 x rs9282541 analysis interaction showed a marginal significance for the HDL-c levels under categorical (Slope difference = 0.51, *p* _interaction_ = 0.03) (data not shown) and continuous (slope difference= 0.064, *p* _interaction_ = 0.064) analysis (Figure 1b). The rs1784042 x rs17120425 interaction was not significant for the HDL-c levels (*p* > 0.05).

### 3.5. Association Analyses between Nutrients with HDL-c Levels

We explored the association between nutrients with HDL-c. The adjusted models showed that per 1 unit of carbohydrates intake, the HDL-c decreased (β = −0.12, 95% CI: −0.19, −0.05), and per 1 unit of total fat and MUFAs intake, the HDL-c increased (β = 0.06, 95% CI: 0.02, 0.09) and (β = 0.05, 95% CI: 0.02, 0.08), respectively. We explored the stratified analysis by sex. In women, we found an association between carbohydrates intake (β = −0.14, 95% CI: −0.22, −0.05), total fat intake (β = 0.09, 95% CI: 0.04, 0.13), MUFAs intake (β = 0.07, 95% CI: 0.03, 0.10), and saturated fat intake (β = 0.04, 95% CI: 0.005, 0.08) with HDL-c levels. Furthermore, in premenopausal women, we observed an association between carbohydrates intake (β = −0.16, 95% CI: −0.29, −0.02), total fat intake (β = 0.11, 95% CI: 0.04, 0.17), MUFAs intake (β = 0.09, 95% CI: 0.03, 0.15), and PUFAs intake (β = 0.06, 95% CI: 0.006, 0.12) with HDL-c levels (Table S4). When we adjusted for the protein intake, the coefficients remained similar. We did not observe a significant association between the nutrients and HDL-c in men and postmenopausal women (Data not shown).

### 3.6. Gene–Diet Interaction with HDL-c Levels

We explored the sex–nutrient interactions on HDL-c and found an interaction with total fat. In women, per each 1 unit of total fat intake, adjusting for protein intake, the HDL-c levels increased (β= 0.09, 95%CI 0.05, 0.14; slope difference= −0.08, *p*_interaction_ = 0.039), while in men, we did not observe significant results. In addition, women with a high consumption of saturated fat intake, adjusting for protein intake, have higher HDL-c levels (OR = 0.66, 95% CI: 0.47–0.94, *p*_interaction_ = 0.014) compared to those with low intakes. We did not observe another sex-nutrient interaction on HDL-c.

When we explored the gene–nutrient interactions in sex-combined analyses, we only found an interaction between saturated fat with rs17120425. In carriers of the rs17120425-A allele, per each 1 unit of saturated fat intake, adjusting for protein intake, the HDL-c levels increased at 0.09 mg/dL (95%CI 0.03, 0.16) (*p*_interaction_ = 0.022). 

**Figure 2:**
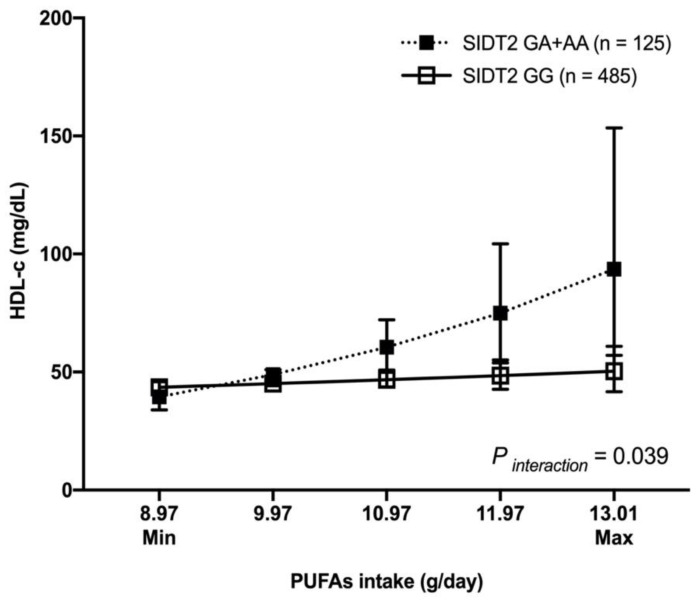
Interaction between *SIDT2*-rs17120425 and PUFAs dietary intake with HDL-c levels in premenopausal women. HDL-c showed as mean, 95% confidence interval. PUFAs intake plotted as the minimum and maximum consumptions in the study population. Model adjusted for age (years), body mass index (Kg/m^2^), physical activity (inactive/active), lipid-lowering medications (no, yes), smoking (no, current, past), diabetes (no, impaired glucose tolerance, yes), and arterial hypertension (SBP ≥ 140 or DBP ≥ 90 or antihypertensive drug); PUFAs: polyunsaturated fatty acids.

We observed a statistically significant gene–nutrient interaction only with the SNP rs17120425 in pre- and postmenopausal women. For example, the premenopausal women carrying the rs17120425 GA + AA genotypes with a high consumption of PUFAs had higher levels of HDL-c compared to those with low consumption (slope difference = 0.18, *p* _interaction_ = 0.039) (Figure 2). 

Additionally, we found through the logistic regression analysis that premenopausal women carrying the genotypes rs17120425 GA + AA and with a high intake of total fat (*p* _interaction_ = 0.044), protein (*p* _interaction_ = 0.049) or MUFAs (*p* _interaction_ = 0.021), seem to be associated with hypoalphalipoproteinemia (Figure S2a–c). Regarding the postmenopausal carriers of the rs17120425 GA + AA genotypes, we observed lower odds of having hyperalphalipoproteinemia when they increase their dietary fiber intake (*p* _interaction_ = 0.043) (Figure S2d). We did not observe significant gene–diet interactions with men. No other gene–nutrient interaction was significantly associated with the study population.

## 4. Discussion

According to epidemiological studies, the Mexican population has one of the highest rates of hypoalphalipoproteinemia [3]. This anomaly is likely due to lifestyle factors and a genetic profile resulting from the ancestry-specific allele combinations derived from the population admixture process [38]. Therefore, analyzing the interaction between the polymorphisms that influence the HDL-c levels in the Mexican population is of great clinical relevance, especially for people with a potential risk of developing metabolic complications from hypoalphalipoproteinemia. To the best of our knowledge, this is the first study analyzing the interaction between three key polymorphisms, rs1784042, rs17120425, and rs9282541, with HDL-c serum levels and their interaction with nutrients in Mexican adults.

In the HWCS population, the prevalence of hypoalphalipoproteinemia was 60.2%, similar to what was previously reported by ENSANUT for Mexican adults (50–60%) [39]. It has been described that several environmental factors contribute to this disturbance, i.e., the high dietary content of carbohydrates and fat in the Mexican diet and the growing incidence of obesity, hypertriglyceridemia, and other metabolic traits [40]. Additionally, results from genetic studies have shown that certain hypoalphalipoproteinemia-susceptible alleles are specific to the Mexican population [41]. In this regard, the MAF of the rs1784042-A in our study population (0.30) is similar to the frequency reported by the 1000 genomes database for the MXL population. The presence of this allele differs around the world, from 0.02 in the African population to 0.42 in Europeans. In contrast, the rs17120425-A and rs9282541-A alleles are found almost exclusively in the American people and are rare or absent in other populations worldwide [38]. The rs9282541-A allele is thought to be preserved in the American population because it provided some adaptative advantage related to energy storage in the past [24]. However, due to changes in environmental factors, such as unhealthy diets and a sedentary lifestyle, this polymorphism may have become a risk factor for metabolic dysregulation [42].

Our results indicated that the rs1784042-A and rs17120425-A alleles were associated with low blood HDL-c levels, consistent with recent reports [13,14]. Current investigations have found that *SIDT2* plays an essential role in the re-localization of cholesterol and the metabolism of glucose and lipids [43,44]. In addition, SIDT2^-/-^ mice models have revealed altered insulin secretion, increased total cholesterol, triglycerides, and lower HDL-c concentrations than wild-type mice [43,45]. These findings suggest that *SIDT2* has a widespread effect on lipid and glucose metabolism and other metabolic traits. Notably, the rs17120425-A allele, compared to rs1784042-A, was strongly associated with high HDL-c levels. Our results agree with León-Mimila et al., who previously proposed a functional role of rs17120425 [14]. They found that the rs17120425-A was associated with high HDL-c and ApoA-1 levels [14]. Nascent HDL particles acquire ApoA molecules, which are essential for their structural and catalytic stability since they are activators that increase circulating HDL-c concentrations [46]. It could explain the most evident effects of rs17120425 on high HDL-c circulation in our analysis.

The *ABCA1* gene codes for the ATP-Binding Cassette Transporter 1, a membrane-associated protein of the superfamily of ATP-binding cassette (ABC) transporters. ABC proteins transport diverse molecules across extra- and intracellular membranes [47]. With cholesterol as its substrate, this protein functions as a cholesterol efflux pump in the cellular lipid removal pathway [48]. Regarding the rs9282541 polymorphism on *ABCA1*, our findings support the association for low HDL-c serum concentrations, similar to what is consistently reported in the Mexican population [5,25,26]. Previously, this variant was linked to a decreased ABCA1 transporter activity (~30%), which plays an essential role in HDL-c biogenesis [26]. Although the genetic association between rs9282541-A and HDL-c concentrations has been documented, the specific metabolic pathways are unknown.

Interestingly, we observed that the effect of the three variants on the HDL-c levels appears to be greater in females than in males. This lack of significant association in the male group was probably due to low statistical power in the analysis (range 0.38–0.51) for the men’s sample size (n = 601). Additionally, previous reports have pointed out that the effect of these variants is stronger in women than in men [13,14,24]. However, the literature has also shown varying results; some studies have associated the rs9282541 variant equally in males and females [25]. Other studies on CVD found a more significant association among males [27]. Possible explanations for these inconsistencies are the sample size, the spurious associations due to population stratification, analyses lacking an adjustment for confounders, or differences in the study design. However, it is also essential to consider certain factors influencing *ABCA1* activity. *ABCA1* is a sex-biased gene present in the liver, expressed at significantly higher levels in females [49]. It has been documented that estrogens increase the *ABCA1* expression [50], and specific dietary components can regulate its expression [51].

Epidemiological studies have consistently pointed out that HDL-c levels are a sexually dimorphic trait; HDL-c levels are 20–25% higher in females than in males, and these differences persist over time [3]. Thus, the genetic variants involved in HDL-c metabolism have also been shown to have a sex-dependent effect. Polymorphisms in *APOA1* (rs1799837) [52], *LIPC* (rs1800588) [16], and *SCARB* (rs838895) [53] represent major determinants of the plasma efflux capacity in females, whereas *ABCA1* (rs2230806) [54] and *APOAII* [55] (rs5082) are determinant predictors in males. These observations are consistent with the fact that sex hormones regulate the genes involved in intravascular HDL-c metabolism, leading to higher HDL-c levels in women than in men [56].

Additionally, many studies have demonstrated that the susceptibility to metabolic alterations is influenced by the interaction between specific genetic variants [57]. In this regard, the rs1784042 x rs9282541 interaction was associated with higher blood HDL-c levels (OR = 0.40, *p* = 1.4 × 10^−4^). Carriers with at least one copy of the rs9282541-A allele and one copy of the rs1784042-A allele had 10.6% higher HDL-c levels than non-carriers (β = 0.106, *p* = 1.4 × 10^−5^). Therefore, the effect of this interaction may be significant in people with an increased risk of CVD and could represent a potential therapeutic strategy against dyslipidemia.

Even though our analysis did not reveal a significant effect of the rs17120425 x rs9282541 interaction, we observed a marginal trend (β = 0.12, *p* = 0.064). A possible explanation for this failure might be the low statistical power due to the low frequency of both alleles in the Mexican population (0.10 for each one). Previous interaction studies between low-frequency genetic variants have been hard to identify [58].

We could speculate that there is a potential biological interaction. SIDT2 is an integral membrane protein associated with cholesterol uptake from the extracellular space into the cell via endocytosis [59]. The binding to cholesterol by the SIDT2 protein is performed specifically by the transmembrane Cholesterol Recognition/interaction Amino acid Consensus (CRAC) domain. A single-point mutation, directed to disrupt the CRAC domains, has been shown to alter cholesterol affinity [60]. Recently, it has been demonstrated that the rs17120425 variant and the CRAC domain are located within the same transmembrane region, suggesting a direct interaction with cholesterol [61], which may affect the circulating plasma concentration of HDL-c [14]. Due to the proximity between rs17120425 and rs1784042 (~2474 bp), both SNPs probably influence the function of the CRAC domain. This finding is supported by an in vitro study, which demonstrated that cells carrying the rs17120425-A allele had higher uptake of the cholesterol analog than the wild-type [14]. Other studies have suggested that the absence of the *SIDT2* gene could block the maturation of autophagosomes and damage the autophagy process at the terminal stage, a critical step in the transfer of exogenous lipids [43,62], which is mainly dependent on *ABCA1* [14]. Several studies have demonstrated that the ABCA1 transporter is regulated by endocytic recycling between the plasma membrane and intracellular compartments [63]. We may infer that inhibiting a pathway responsible for endocytosis and degradation of the ABCA1 protein could influence its abundance and exposure at the cell’s surface and cholesterol efflux, affecting the HDL-c plasma levels. However, further studies are needed to confirm the metabolic pathways involved.

Concerning the gene–nutrient interaction, we observed that pre- or postmenopausal women carriers of the rs17120425-A allele had higher blood HDL-c levels when they consumed a diet rich in fat, protein, MUFAs, PUFAs, or fiber. To the best of our knowledge, this is the first study suggesting an interaction between *SIDT2* and nutrients with HDL-c concentrations. However, current studies have also observed significant interactions between the genetic variants involved in HDL-c metabolism and nutritional factors on HDL-c levels [64,65,66,67]. Supporting our results, earlier studies have shown that a high-fat intake can increase the HDL-c concentrations through more substrate provided in the chylomicron-derived triglycerides and increased lipoprotein lipase (LPL) activity. LPL activity is associated with HDL-c concentrations, with greater activity and, thus, catabolism of triglyceride-rich lipoproteins, resulting in elevated HDL-c concentrations [68]. However, the effects of different fatty acids on serum HDL-c concentrations are inconsistent. A previous investigation demonstrated that serum HDL-c concentrations are low in people consuming high saturated fatty acids (SFAs) and are unaffected by the intake of either MUFAs or PUFAs [69]. However, an early controlled-feeding trial study suggested that older women who consume higher amounts of dietary MUFAs have a more favorable effect on metabolic profiles, including HDL-c levels, compared to those who consume a diet rich in PUFAs [65]. In addition, observational studies have also explored the interaction between dietary factors on dyslipidemias. Those results support our findings that subjects with higher daily protein and fiber intake have elevated serum HDL-c levels [70,71].

A sex-specific gene–diet interaction was observed in the current analysis, particularly in premenopausal and postmenopausal women. Although the cause of sex-related differences is not fully understood, it has been reported that estrogens have a key effect in premenopausal women by increasing HDL-c concentrations [72]. The literature indicates that sex hormones modulate the intrinsic capacity of circulating HDL-c particles to mediate the cellular efflux of free cholesterol and distinct quantitative and qualitative features of HDL-c particles between men and women. HDL-c is a heterogeneous group of lipoprotein particles that may be classified by decreasing size in HDL2b, HDL2a, HDL3a, HDL3b, and HDL3c. These HDL-c subclasses differ in apolipoprotein and lipid composition, size, density, and charge [73]. Compared to large HDL-c particles, small HDL-c particles (HDL3a-c) are better cholesterol acceptors [74], exhibit more antioxidative activity [75], and have a higher capacity to inhibit the expression of adhesion molecules [76]. This finding highlights the importance of considering the sex-dependent effects of gene variants on serum lipid levels.

On the other hand, we did not find significant interactions between the rs9282541 and nutrients in our study population. This is contrary to what was previously reported in two independent studies, which pointed out an inverse correlation between carbohydrate intake and HDL-c concentrations in women bearing the rs9282541-A allele and a significant gene–diet interaction only in premenopausal women [77,78]. This discrepancy could be because a residual model that indirectly adjusts for total energy was used in this study.

This research provided evidence that the inter-individual variability of HDL-c levels is related to the interaction between genetic polymorphisms and nutrient intake in the Mexican population. Our results open the door for identifying potential nutrigenetic recommendations that consider the established interactions between genetic polymorphism and their influence on nutrient intake, which could explain the inter-individual variation in hypoalphalipoproteinemia and provide possible strategies for personalized treatment. It should be noted that the findings of this study can be extrapolated to individuals living in urban areas of Mexico; therefore, its application should be taken with caution for other populations.

The strengths of the current study are described below. First, this analysis included a large sample of individuals compared to other observational studies. Second, the rigorous statistical analysis adjusted for potential confounding covariables, reducing the risk of spurious associations. Third, this is the first effort to understand the effect of the gene–gene and gene–diet interactions involving the *SIDT2* and *ABCA1* genes on HDL-c concentrations in the Mexican population. Nevertheless, this study has some limitations. First, the scarce information about other genetic variants and environmental factors that may affect HDL-c concentrations. Second, the FFQ data were self-reported; this can be subject to random measurement errors, leading to underestimating the association. However, the FFQ was designed and validated by the National Institute of Public Health in Mexico to assess long-term exposure to different nutrients to study their potential role as risk factors for chronic disease. Third, we did not consider estrogen levels, which have been reported to influence lipid levels. Fourth, despite a sufficient overall sample size, the lack of statistical significance for some covariates could reflect the low statistical power in the men’s group (n = 601). Fifth, due to the low frequency of the SNPs analyzed in the interaction model, it is necessary to increase the sample size to have adequate statistical power. Sixth, we did not adjust for multiple comparisons due to the effect size of the interactions found and the limited statistical power (0.63–0.74). However, it must be considered that the adjustment by multiple tests controls the global type-I error but considerably inflates the type-II error [79]. Additional studies in other populations and ethnicities, as well as functional experiment studies, are required to support our findings and explain the mechanisms involved in the interaction of genetic variants and nutrients in HDL-c concentrations.

## 5. Conclusions

To the best of our knowledge, this is the first study to report the interaction between the *SIDT2* and *ABCA1* susceptibility gene variants and their association with nutrient intake, contributing to an understanding of the genetic architecture of serum HDL-c concentrations in admixed populations. Understanding the influence of nutrigenetic interactions on hypoalphalipoproteinemia can aid in developing and implementing personalized dietary strategies to improve the health of the Mexican population.

## Figures and Tables

**Table 1:** Demographic and clinical characteristics of the Health Workers Cohort Study.

Characteristics	Men	Women **	*p* *
	n = 601	n = 1381	
Age, years ^a^	46(36–56)	54(43–63)	<0.001
Body mass index ^a^, Kg/m^2^	26.5(24.1–29.0)	26.8(24.0–30.1)	0.161
LDL-c ^a^, mg/dL	115.5(96.7–143.6)	121(99.1–146.6)	0.006
HDL-c ^a^, mg/dL	39(34–46)	46(39–54)	<0.001
Low HDL-c (%)	51.8	63.9	<0.001
Triglycerides ^a^, mg/dL	169(118–247)	151(109–201)	<0.001
Total cholesterol ^a^, mg/dL	192(168–221)	199(172–227)	0.0002
Dietary intake			
Energy ^a^, kcal/day	1957(1458–2556)	1687(1255–2227)	<0.001
Carbohydrates ^a^, g/day	312(237–411)	278(204–372)	<0.001
Protein ^a^, g/day	59.5(44.2–78.3)	52.2(38.1–71.0)	<0.001
Total fat ^a^, g/day	43.2(30.7–61.9)	37.5(27.0–52.7)	<0.001
Monounsaturated fat ^a^, g/day	17.7(12.9–25.0)	15.6(11.2–22.3)	<0.001
Polyunsaturated fat ^a^, g/day	8.6(6.4–12.1)	7.7(5.5–10.7)	<0.001
Saturated fat ^a^, g/day	15.2(10.2–22.0)	12.8(8.8–18.9)	<0.001
SIDT2-rs1784042, n (%)			
GG	298 (50.6)	690 (50.5)	0.974
GA + AA	291 (49.4)	676 (49.5)	0.974
SIDT2-rs17120425, n (%)			
GG	487 (93.0)	1112 (81.4)	0.414
GA + AA	100 (17.0)	254 (18.6)	0.414
ABCA1-rs9282541, n (%)			
GG	486 (81.3)	1106 (80.3)	0.623
GA + AA	112 (18.7)	271 (19.7)	0.623

^a^ Median (P25-P75). * Mann–Whitney *U* test for continuous and the immediate two-sample proportions test for categorical variables were used. ** Premenopausal women n = 616 (44.6%), Postmenopausal women n = 765 (55.5%).

**Table 2:** Characteristics of the study population categorized by genotype.

Characteristics	*SIDT2*-rs1784042			*SIDT2*-rs17120425			*ABCA1*-rs9282541		
	GG	GA + AA	*p* *	GG	GA + AA	*p* *	GG	GA + AA	*p* *
	n = 988 (50.6%)	n = 967 (49.4%)		n = 1599 (81.9%)	n = 354 (18.1%)		n = 1592 (80.6%)	n = 383 (19.4%)	
Age, years ^a^	51 (40–61)	52 (40–62)	0.347	52 (40–62)	52 (41–60)	0.493	52 (40–62)	52 (42–62)	0.296
Women n (%)	690 (69.8)	676 (69.9)	0.967	1112 (69.5)	254 (71.7)	0.490	1106 (69.5)	271 (70.8)	0.676
Body mass index ^a^, Kg/m^2^	26.9 (24.2–30.0)	26.5 (23.9–29.4)	0.070	26.7 (24.1–29.7)	26.7 (23.8–30.0)	0.751	26.7 (23.9–29.7)	26.9 (24.3–30.1)	0.266
LDL-c ^a^, mg/dL	117.8 (97.9–142)	122 (100–148)	0.004	119.9 (98–144.7)	121 (100–148)	0.349	119.5 (98–145)	121 (99.7–146.7)	0.580
HDL-c ^a^, mg/dL	43.1 (36.5–50.6)	44.8 (38–52.8)	0.002	43 (36.8–50.8)	47 (40–55)	<0.001	44.1 (38–52)	41.4 (35.2–49.3)	<0.001
HDL-c categories, n (%)									
≥40 men and ≥50 women	359 (36.3)	421 (43.5)	0.041	590 (36.9)	186 (52.5)	<0.001	655 (41.1)	128 (33.4)	0.102
<40 men and <50 women	629 (63.7)	545 (56.5)	0.011	1009 (63.1)	168 (47.5)	<0.001	937 (58.9)	255 (66.6)	0.025
Triglycerides ^a^, mg/dL	159 (116–218)	152 (109–204)	0.018	158 (112–210)	147 (109–200)	0.182	156 (111.5–210.5)	154 (113–201)	0.597
Total cholesterol^a^, mg/dL	195 (170–225)	198 (171–226)	0.223	195 (170–225)	199 (178–228)	0.025	197 (171–225)	194 (166–228)	0.390

* For categorical variables, the immediate two-sample proportions test was used, and for continuous variables, we used the Mann–Whitney *U* test. ^a^ Median (P25- P75). Low HDL-c: <40 mg/dL in men and <50 mg/dL in women.

**Table 3:** Association between rs1784042, rs17120425, and rs9282541 variants and low HDL-c.

Gene SNP	Model	Normal HDL-c n(%)	Low HDL-c n(%)	Adjusted Model ^1^OR (95% CI)	*p*	Adjusted Model ^2^OR (95% CI)	*p*
*SIDT2* rs1784042	Dominant GG	359 (46.0)	629 (53.5)	Ref		Ref	
	GA + AA	421 (54.0)	546 (46.5)	0.74 (0.61–0.90)	0.002	0.75 (0.62–0.91)	0.004
	Additive	780 (39.9)	1175 (60.1)	0.77 (0.66–0.89)	0.001	0.77 (0.66–0.90)	0.001
*SIDT2* rs17120425	Dominant GG	590 (76.0)	1009 (85.7)	Ref		Ref	
	GA + AA	186 (24.0)	168 (14.3)	0.48 (0.38–0.62)	7.4 × 10^−9^	0.48 (0.37–0.62)	9.6 × 10^−9^
	Additive	776 (39.7)	1177 (60.3)	0.53 (0.42–0.66)	4.1 × 10^−8^	0.52 (0.42–0.66)	5.4 × 10^−8^
*ABCA1* rs9282541	Dominant GG	655 (83.7)	937 (78.6)	Ref		Ref	
	GA + AA	128 (16.3)	255 (21.4)	1.38 (1.08–1.76)	0.011	1.41 (1.09–1.81)	0.008
	Additive	783 (39.7)	1192 (60.3)	1.32 (1.05–1.66)	0.016	1.34 (1.06–1.69)	0.013

Low HDL-c levels: <40 mg/dL in men and <50 mg/dL in women. ^1^ Model adjusted for age (years), sex, body mass index (Kg/m^2^), physical activity (inactive/active), and lipid-lowering medications (no, yes). ^2^ Model additionally adjusted for smoking (no, current, past), diabetes (no, impaired glucose tolerance, yes), and hypertension (SBP ≥ 140 mm/Hg or DBP ≥ 90 mm/Hg or antihypertensive drug); OR: odds ratio, CI: confidence interval.

**Table 4:** Association between rs1784042, rs17120425, and rs9282541 polymorphisms and HDL-c stratified by sex.

		Women (n = 1381)						Men (n = 601)					
Gene SNP	Model	Normal HDL-c n(%)	Low HDL-c n(%)	Model ^1^ OR (95%CI)	*p*	Model ^2^ OR (95%CI)	*p*	Normal HDL-c n(%)	Low HDL-cn(%)	Model ^1^ OR (95%CI)	*p*	Model ^2^ OR (95%CI)	*p*
*SIDT2* rs1784042	Dominant GG	220(44.5)	470(53.9)	Ref		Ref		139(48.6)	159(52.5)	Ref		Ref	
	GA + AA	274(55.5)	402(46.1)	0.70 (0.55–0.88)	0.003	0.71 (0.56–0.90)	0.005	147(51.4)	144(47.5)	0.83 (0.59–1.16)	0.278	0.84 (0.60–1.17)	0.312
	Additive	494(36.2)	872(63.8)	0.76 (0.63–0.90)	0.002	0.76 (0.63–0.92)	0.004	286(48.6)	303(51.4)	0.81 (0.61–1.06)	0.119	0.81 (0.61–1.06)	0.128
*SIDT2* rs17120425	Dominant GG	365(74.2)	747(85.5)	Ref		Ref		225(79.2)	262(86.5)	Ref		Ref	
	GA + AA	127(25.8)	127(14.5)	0.48 (0.36–0.64)	5.3 × 10^−7^	0.43 (0.32–0.59)	6.0 × 10^−8^	59(20.8)	41(13.5)	0.56 (0.36–0.88)	0.012	0.59 (0.37–0.93)	0.022
	Additive	492(36.0)	874(64.0)	0.53 (0.40–0.69)	2.5 × 10^−6^	0.48 (0.37–0.64)	4.3 × 10^−7^	284(48.4)	303(51.6)	0.59 (0.39–0.88)	0.012	0.61 (0.40–0.93)	0.023
*ABCA1* rs9282541	Dominant GG	413(83.3)	693(78.7)	Ref		Ref		242(84.3)	244(78.5)	Ref		Ref	
	GA + AA	83(16.7)	188(21.3)	1.37 (1.01–1.85)	0.042	1.41 (1.03–1.93)	0.029	45(15.7)	67(21.5)	1.42 (0.93–2.19)	0.103	1.44 (0.93–2.26)	0.103
	Additive	496(36.0)	881(64.0)	1.30 (0.99–1.72)	0.053	1.34 (1.01–1.77)	0.040	287(48.0)	311(52.0)	1.37 (0.92–2.06)	0.123	1.39 (0.92–2.12)	0.116

Low HDL-c: <40 mg/dL in men and <50 mg/dL in women. ^1^ Model adjusted for age (years), body mass index (Kg/m^2^), physical activity (inactive/active), and lipid-lowering medications (no, yes). ^2^ Model additionally adjusted for smoking (no, current, past), diabetes (no, impaired glucose tolerance, yes), and hypertension (SBP ≥140 mm/Hg or DBP ≥90 mm/Hg or antihypertensive drug); OR: odds ratio, CI: confidence interval.

## Data Availability

The datasets analyzed in this study are available from the corresponding author upon reasonable request.

## Supplementary Materials

The following supporting information can be downloaded at: https://www.mdpi.com/article/10.3390/nu15020370/s1, Figure S1: Interaction between *SIDT2*-rs17120425 and nutrients on HDL-c levels in premenopausal (a–c) and postmenopausal (d) women; Table S1: Association between rs1784042, rs17120425, and rs9282541 polymorphisms and HDL-c; Table S2: Association between rs1784042, rs17120425, and rs9282541 polymorphisms and HDL-c, by sex; Table S3. Conditional Analysis; Table S4: Association between nutrients with HDL-c levels.
