# Supplementary material for: Interaction between SIDT2 and ABCA1 Variants with Nutrients on HDL-c Levels in Mexican Adults
Source: Nutrients. 2023 Jan 11;15(2):370. doi: 10.3390/nu15020370 (PMC9861312; doi:10.3390/nu15020370)
Supplement: Supplementary file 1 [file nutrients-15-00370-s001.zip › nutrients-1952300-supplementary.pdf]

Table S1. Association between rs1784042, rs17120425 and rs9282541 polymorphisms and HDL-c.

Gene	SNP	Model	Adjusted Model [1] β (95% CI)	*p*	Adjusted Model [2] β (95% CI)	*p*
SIDT2	rs1784042	Dominant GG	Ref		Ref	
		GA+AA	0.03 (0.01, 0.05)	0.003	0.03 (0.01, 0.05)	0.004
		Additive	0.03 (0.01, 0.04)	3×10^{-4}	0.03 (0.01, 0.04)	5×10^{-4}
SIDT2	rs17120425	Dominant GG	Ref		Ref	
		GA+AA	0.08 (0.05, 0.10)	2.5×10^{-8}	0.07 (0.05, 0.10)	7.8×10^{-8}
		Additive	0.07 (0.04, 0.09)	8.5×10^{-8}	0.07 (0.04, 0.09)	2.5×10^{-7}
ABCA1	rs9282541	Dominant GG	Ref		Ref	
		GA+AA	−0.06 (−0.08, −0.03)	6.3×10^{-6}	−0.06 (−0.09, −0.03)	4.8×10^{-6}
		Additive	−0.05 (−0.07, −0.02)	1.6×10^{-5}	−0.05 (−0.07, −0.02)	1.3×10^{-5}

Low HDL-c: <40 mg/dL in men and <50 mg/dL in women. [1] Model adjusted for age (years), sex, body mass index (Kg/m^2), physical activity (inactive/active) and lipid-lowering medications (no, yes). [2] Model additionally adjusted for smoking (no, current, past), diabetes (no, impaired glucose tolerance, yes) and hypertension (SBP ≥140 mm/Hg or DBP ≥90 mm/Hg or antihypertensive drug); * Carriers of the ancestral genotype GG; β: regression coefficient, CI: confidence interval.

Table S2. Association between rs1784042, rs17120425 and rs9282541 polymorphisms and HDL-c, by sex.

Gene SNP	Model	Women (n = 1,381) Adjusted Model[1] β (95% CI)	*p*	Women Adjusted Model[2] β (95% CI)	*p*	Men (n = 601) Adjusted Model[1] β (95% CI)	*p*	Men Adjusted Model[2] β (95% CI)	*p*
SIDT2 rs1784042	Dominant GG	Ref		Ref		Ref		Ref	
	GA+AA	0.03 (0.005, 0.06)	0.016	0.03 (0.004, 0.06)	0.020	0.03 (−0.002, 0.07)	0.068	0.03 (−0.006, 0.07)	0.104
	Additive	0.03 (0.008, 0.05)	0.005	0.03 (0.007, 0.05)	0.006	0.03 (0.004−0.06)	0.024	0.03 (0.001−0.06)	0.039
SIDT2 rs17120425	Dominant GG	Ref		Ref		Ref		Ref	
	GA+AA	0.08 (0.05, 0.11)	4.8×10^{-7}	0.08 (0.05, 0.12)	1.0×10^{-6}	0.06 (0.01, 0.10)	0.012	0.06 (0.009, 0.11)	0.020
	Additive	0.07 (0.04, 0.10)	1.2×10^{-6}	0.07 (0.04, 0.10)	2.5×10^{-6}	0.05 (0.01, 0.09)	0.016	0.05 (0.005, 0.09)	0.026
ABCA1 rs9282541	Dominant GG	Ref		Ref		Ref		Ref	
	GA+AA	−0.06 (−0.09, −0.03)	5×10^{-5}	−0.07 (−0.10, −0.03)	3×10^{-5}	−0.05 (−0.09, −0.006)	0.026	−0.05 (−0.10, −0.006)	0.026
	Additive	−0.06 (−0.08, −0.03)	1.2×10^{-4}	−0.06 (−0.08, −0.03)	8.4×10^{-5}	−0.05 (−0.09, −0.003)	0.034	−0.05 (−0.09, −0.004)	0.033

Low HDL-c: <40 mg/dL in men and <50 mg/dL in women. [1] Model adjusted for age (years), body mass index (Kg/m^2), physical activity (inactive/active) and lipid-lowering medications (no, yes). [2] Model additionally adjusted for smoking (no, current, past), diabetes (no, impaired glucose tolerance, yes) and hypertension (SBP ≥140 mm/Hg or DBP ≥90 mm/Hg or antihypertensive drug); β: regression coefficient, CI: confidence interval.

Table S3. Conditional Analysis.

Gene	SNP	Conditioned by	OR (95% CI)	*p*	β (95% CI)	*p*
SIDT2	rs1784042	rs17120425	0.941 (0.760–1.165)	0.578	0.009 (−0.014–0.031)	0.463
		rs9282541	0.762 (0.627–0.927)	0.006	0.027 (0.006–0.048)	0.011
SIDT2	rs17120425	rs1784042	0.485 (0.369–0.639)	$2.44x10^{-7}$	0.072 (0.042–0.102)	2.09×10^{-6}
		rs9282541	0.473 (0.367–0.609)	$6.27x10^{-9}$	0.076 (0.049–0.104)	3.90×10^{-8}
ABCA1	rs9282541	rs1784042	1.415 (1.095–1.829)	0.008	−0.064 (−0.906–0.037)	3.14×10^{-6}
		rs17120425	1.470 (1.136–1.904)	0.003	−0.066 (−0.093–0.040)	1.11×10^{-6}

Model adjusted for age (years), sex, body mass index (kg/m^2), physical activity (inactive/active), lipid-lowering medications (no, yes), smoking (no, current, past), T2D (no, impaired glucose tolerance, yes) and hypertension (SBP ≥140 mm/Hg or DBP ≥90 mm/Hg or antihypertensive drug); OR: Odd ratio, CI: confidence interval.

Table S4. Association between nutrients with HDL-c levels.

Nutrient	Total (n = 1,982) Adjusted Model[1] β (95% CI)	*p*	Women (n = 1,381) Adjusted Model[2] β (95% CI)	*p*	Premenopausal women (n = 616) Adjusted Model[2] β (95% CI)	*p*
Carbohydrates, g/day	−0.12 (−0.19, −0.05)	0.001	−0.14 (−0.22, −0.05)	0.002	−0.16 (−0.29, −0.02)	0.021
Protein, g/day	0.002 (−0.04, 0.06)	0.658	0.01 (−0.04, 0.07)	0.622	−0.03 (−0.12, 0.06)	0.480
Fiber, g/day	−0.008 (−0.03, 0.02)	0.492	−0.02 (−0.05, 0.005)	0.108	−0.04 (−0.08, 0.005)	0.088
Total fat, g/day	0.06 (0.02, 0.09)	0.002	0.09 (0.04, 0.13)	$1.6x10^{-4}$	0.11 (0.04, 0.17)	0.001
Monounsaturated fat, g/day	0.05 (0.02, 0.08)	0.001	0.07 (0.03, 0.10)	0.001	0.09 (0.03, 0.15)	0.002
Polyunsaturated fat, g/day	0.02 (−0.01, 0.05)	0.190	0.03 (−0.009, 0.07)	0.147	0.06 (0.006, 0.12)	0.029
Saturated fat, g/day	0.02 (−0.006, 0.05)	0.118	0.04 (0.005, 0.08)	0.024	0.04 (−0.02, 0.09)	0.199

[1] Model adjusted for age (years), sex, body mass index (Kg/m^2), physical activity (inactive/active), lipid-lowering medications (no, yes), smoking (no, current, past), diabetes (no, impaired glucose tolerance, yes), and hypertension (SBP ≥140 mm/Hg or DBP ≥90 mm/Hg or antihypertensive drug). [2]Same as Model 1, but without sex adjustment. β: regression coefficient, CI: confidence interval.

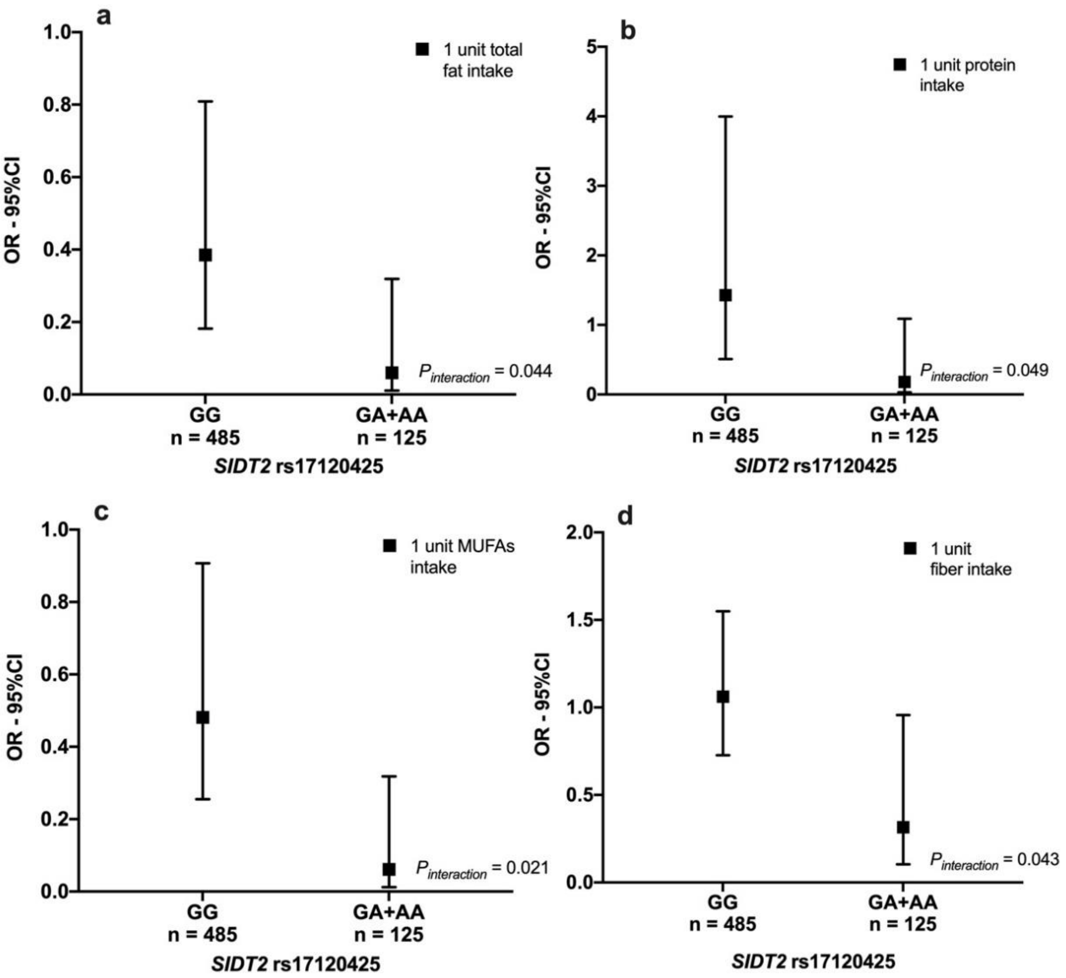

Figure S1. Interaction between *SIDT2* rs17120425 and dietary nutrients on HDL-c levels in premenopausal (**A**–**C**) and postmenopausal (**D**) women. Interactions between rs17120425 variant with (**A**) total fat, (**B**) protein, (**C**) MUFAs, (**D**) PUFAs and E) fiber dietary. Model adjusted for age (years), body mass index (Kg/m^2), physical activity (inactive/active), lipid-lowering medications (no, yes), smoking (no, current, past), diabetes (no, impaired glucose tolerance, yes) and hypertension (SBP ≥140 or DBP ≥90 or antihypertensive drug); MUFAs: Monounsaturated fatty acids; PUFAs: Polyunsaturated fatty acids.
